# Health taxes in Indonesia: a review of policy debates on the tobacco, alcoholic beverages and sugar-sweetened beverage taxes in the media

**DOI:** 10.1136/bmjgh-2023-012042

**Published:** 2023-10-09

**Authors:** Abdillah Ahsan, Nadira Amalia, Krisna Puji Rahmayanti, Nadhila Adani, Nur Hadi Wiyono, Althof Endawansa, Maulida Gadis Utami, Adela Miranti Yuniar

**Affiliations:** 1 Department of Economics, Faculty of Economics and Business, University of Indonesia, Depok, West Java, Indonesia; 2 Demographic Institute, Faculty of Economics and Business, University of Indonesia, Depok, Indonesia; 3 Department of Public Administration, Faculty of Administrative Science, University of Indonesia, Depok, Indonesia; 4 Center for Research in Islamic Economics and Business, Universitas Indonesia Faculty of Economics and Business, Depok, West Java, Indonesia

**Keywords:** health policy, health economics, public health

## Abstract

**Introduction:**

One of the WHO’s ‘best buys’ in controlling non-communicable diseases and their risk factors is to impose health taxes. While the Indonesian political process inhibits the implementation of health tax policy, studies to discuss the issue remain limited.

**Methods:**

We employed media analysis to document health tax policy dynamics, for example, the changes in policy timeline and key actors’ statements. We conducted an article search in the Open-Source Intelligence database using appropriate terminology on three commodities, for example, tobacco, alcoholic beverages and sugar-sweetened beverages (SSB).

**Results:**

Throughout the 15 years of implementation (2007–2022), tobacco has received the most policy attention compared with the other two commodities. This is mainly related to the increasing tariff and reforming the tax structure. As Indonesia is a Muslim-majority country, alcohol consumption is low, and a tax on alcoholic beverages was nearly unchanging and lacked media coverage. Ministry of Finance (MoF) officials are key opinion leaders often cited in the media for health taxes. MoF’s support for health taxes is important to pass and implement health taxes. While SSB taxation is emerging, key opinion leaders’ media statements imply policy contestation, leading to delayed implementation. The policy debates on tobacco taxation implied election years as a major challenge for health tax passages. During the political years, anti-health tax arguments emerged from politicians. While the political contestation on SSB concluded that accentuating the health tax arguments in favour of public health generates the strongest opposition against taxation from the industry.

**Conclusions:**

Politics of tobacco tax implementation are complex—compared with the other two commodities. The political context drives the divided views among policy-makers. Policy recommendations include generating public allies with key religious opinion leaders, continuing capacity building for politicians and Ministry of Health, and generating evidence-based arguments in favour of public health for MoF.

**WHAT IS ALREADY KNOWN ON THIS TOPIC**

- The media coverage against policy debate on tobacco and e-cigarette taxation in Indonesia favours economic interests over health concerns.
- Political interests drive and limit tobacco tax policy-making, resulting in poorly conceived policies and unmet policy objectives.

**WHAT THIS STUDY ADDS**

- We reviewed and analysed the main arguments on health taxes (including sugar-sweetened beverage (SSB) and alcoholic beverages in addition to tobacco) in Indonesia’s national media. This was not discussed in previous literature for the Indonesian context, which majorly focused on tobacco.
- Tobacco tax policies evolved significantly compared with those for SSB and alcoholic beverages. The 2018–2019 was an important political year and showcased the importance of media framing.

**HOW THIS STUDY MIGHT AFFECT RESEARCH, PRACTICE OR POLICY**

- The Ministry of Finance’s support for health taxes is a large determinant in passing and expanding health taxes. Gathering evidence in favour of public health and revenue generation to secure the Ministry of Finance’s support could be the most important strategy to advance health taxes in Indonesia.

## Introduction

Low-income and middle-income countries (LMICs) bear a disproportionate burden of non-communicable diseases (NCDs) that cost over US$2 trillion annually.^1 2^ The economic burden is primarily related to the loss due to disability-adjusted life-years (DALYs) and financial distress of catastrophic major NCDs.^3 4^ The high cost of catastrophic diseases requires extra funding and, to an extent, could potentially generate a health financing deficit, including in Indonesia.^5 6^

Jaminan Kesehatan Nasional (JKN), Indonesia’s national health insurance, has been challenged by fiscal sustainability, partly due to a budget deficit.^7^ One of the significant costs is major catastrophic diseases (including stroke, ischaemic heart diseases, chronic kidney diseases, stroke, cancer, cirrhosis and chronic liver diseases, thalassaemia, leukaemia and haemophilia), which accounted for 25% of total JKN claims in 2020.^8^ The risk factors attributable to unhealthy diet and consumption (eg, tobacco, high blood pressure, dietary risks, high fasting plasma glucose and high body mass index) are increasingly contributing to DALYs.^9^ Children’s diabetes cases have increased 70 times from 2010.^10^ These diseases are attributed to certain commodities’ consumption, including cigarettes, alcohol and sugar-sweetened beverages (SSBS).^11–13^ Indonesia has the largest market for cigarette consumption among male adults (reaching 71% in 2020).^14^ In addition, smoking prevalence among adolescents continued to increase.^15^ Therefore, controlling the consumption of these commodities is important to prevent NCDs’ prevalence.

One of the WHO’s best buys in controlling NCDs and their risk factors is to impose health taxes.^16^ This measure will increase price, reduce the affordability of harmful commodities and create additional government revenue, which could be allocated to finance the healthcare sectors.^17^ This strategy is a successful measure in many LMICs.^18–22^ Health taxes have been widely successful in providing sustainable health financing.^23^ Given the burden of disease and existing trends, Indonesia should improve existing health taxes for tobacco and alcohol and implement a new health tax for SSB; however, there are political challenges.

Compared with other ASEAN (The Association of Southeast Asian Nations) countries, Indonesia has the least number of taxable goods^24^ with only three commodities currently being taxed (eg, tobacco products, ethyl alcohol and beverages containing ethyl alcohol) and two under government review (eg, SSB and plastic packaging taxes). The regulatory framework in Indonesia is highly rigid, which, combined with the lack of technical guidelines on the law implementation, may result in disagreements and conflicting interests.

In addition, industries interfere in policy-making. Learning from tobacco tax experiences, the large-scale industries are misleadingly using the welfare of vulnerable groups (eg, farmers and industry workers) to argue against tobacco tax. This is covered widely in the media.^25^ The media framing for strong opposition from large industries has divided public’s opinion. Politicians and government officials are also constantly in disputes about tobacco tax policy.^26^ Furthermore, their support for economic interests (than public health) is more profound.^27^

Drawing from tobacco tax experiences, health tax implementation encounter challenges due to conflicting interests that can be augmented by the industry’s interference through the media. The government’s agenda to extend or advance taxable commodities may face the same issue. The media framing builds the narrative on public policy debates, and ignoring the media effects could undermine the policy agenda-setting.^28^ Hence, capturing media coverage on health tax would generate valuable input for health tax passage and implementation. While the nature of health tax can be complex, studies have solely focused on tobacco taxation.^26 27^ This study voids this gap by extending taxable commodities to include alcohol and SSB.

## Methods

This study is exploratory and qualitative to generate government information (meaning policy-makers working in government ministries and agencies), politicians (meaning elected officials), industry and public perspectives.^29^ Media framing plays an active role in framing public policy issues.^30^ We conducted media analysis as the data collection method to allow a broad analysis of different elements (eg, statements, actors, news tone).^31^ The media analyses helped us to understand the policy dynamic and generate critical timeline of health tax policies, providing leading statements of key actors at the national level and generate a better understanding of the media framing strategy. We focused on three excisable commodities: tobacco products, alcoholic beverages and SSB.

### Search strategy

We searched for news articles in the Open-Source Intelligence database from 4 July 2022 to 7 July 2022, using relevant terms (see the next paragraph). Only Indonesian language news articles were included, which is translated into English for the quotations presented in this article. The entire queries were also in Indonesian. Articles were selected from 9676 national online and 328 national printed media. The articles displayed are from 1 January 2018 to 3 July 2022. The chosen article is a news story with at least one paragraph about tobacco products, alcoholic beverages or SSB tax. Duplicated articles were removed (news covering tobacco, SSB or alcoholic beverages in a single article did not count as a duplicate—we focused on the statement instead).

For the tobacco products articles, we used the following queries: ((“tax simplification “~3 OR “tariff structure simplification” OR “tariff simplification” OR “tax harmonization” OR “tax increase “~3 OR “tax increase “~3 OR “increase in taxes “~3 OR “increase in taxes “~3 OR “increase in taxes” OR “increase in cigarette prices”) AND (“cigarettes” OR “tobacco” OR “E-cigarettes” ~3 OR “e-cigarettes” ~3 OR “vape” OR “vaporizer” OR “vapor”)) NOT “corruption.” The NOT “corruption” query is used to filter articles relevant to tobacco excise and make the dataset more manageable.

To find articles that directly mention the tax duty on alcoholic beverages and SSB, we used the queries (“alcoholic beverages tax” OR” alcohol tax”) and (“sugar-sweetened-beverages tax” OR” sweetened beverages tax” OR “SSB tax” OR “sugary drink tax”). We used the following queries to find articles that mention tax and the alcoholic beverages or tax and SSB separately: ((“tax”) AND (“alcoholic beverages” OR “alcohol”)) and ((“tax”) AND (“sugar-sweetened-beverages” OR” sweetened beverages” OR “SSB” OR “sugary drink”).

### Content analysis

All articles were analysed with NVivo V.12 software. We used content analysis to gather information on media sentiment and framing against each commodity, following content analysis structure on tobacco and SSB media coverage in other countries.^32 33^ The news tone or sentiment illustrates the message that news conveyed towards the issue (eg, tobacco, alcohol or SSB tax) that has the potential lead public opinion. We identified the key opinion leaders (most frequently cited actors by the number of statements) and the sentiment and topic throughout the time (to identify the dynamics of the news coverage against the taxable commodity).

**Figure 1:**
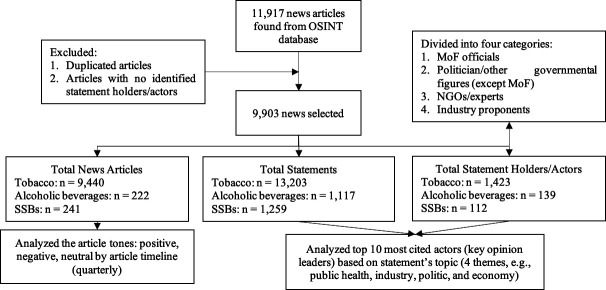
Media analysis flow chart. MoF, Ministry of Finance; OSINT, Open-Source Intelligence; SSBs, sugar-sweetened beverages. NGOs is a nonprofit organization that operates independently of any government whose purpose is to address a public health issue.

We analysed the key opinion leaders’ statements across four themes, for example (1) public health (keywords: health, diseases, NCD, obesity, overweight, control); (2) industry (keywords: industry, company, sales, association, farmers and enterprises); (3) politics (keywords: poverty, political party, budgeting, regulation and legislation) and (4) economy (keywords: tax (but excluding keywords of other themes) and revenue). We also divided the actors into four categories, for example, Ministry of Finance (MoF) officials, politicians or other governmental figures (except MoF) (eg, other ministerial figures or local government), NGOs (Non Governmental Organizations)/experts and industry proponents (see figure 1). NAd and AE; KPR and AMY; and NAm and MGU each coded the statements for tobacco, alcoholic beverages and SSB, respectively. NHW and NAm ensured the keyword’s appropriateness. Disagreement among team members during the coding process was discussed together accordingly.

## Results

To organise the results, we first discussed policy milestones to understand changes in health tax policies over time. Second, we presented findings by timeline, news tone, cited actors and statements’ theme. Finally, we discussed overall findings for all commodities and compared and contrasted findings for each.

### Health tax policy dynamics in Indonesia

Since 2007, tobacco taxes have evolved more than the other two commodities. This is mainly related to increasing tariffs, tax simplification and regional tax arrangements. Though taxing tobacco products and alcoholic beverages started at the same phase after the first version of excise law in 1995, the policies on alcohol tax are nearly unchanging. There are significantly fewer consumers of alcoholic beverages—compared with tobacco products (average of 3.3% for alcohol compared with 29.3% for tobacco, nationally),^34^ since Indonesia is a Muslim-majority country and alcohol consumption is low. Alcohol consumption is only common in some Muslim-minority provinces. Whereas the SSB tax is still under review, there have been some pros and cons (see table 1), leading to a delay in its implementation since it was first proposed in 2019. Table 2 summarises the health tax policy dynamics.

**Table 1:** Summary of selected quotations by year and statements’ position

Year	In support of health taxes	Against health taxes
2018	On alcoholic beverages tax ‘…*although there was no increase in [alcoholic beverages] production for four years, there was also no increase in tax duty. Therefore, the tax payments to factory turnover ratio had decreased in the same period*…’ (NDH, MoF officials)	On tobacco tax ‘…*the policy had been made based on the aspirations of retailers, industry, and tobacco farmers*…’ (MM, politician) On alcoholic beverages tax ‘…*since 2014 to 2017 and also 2018, the domestic industry for (alcoholic beverages) type A or beer has a steady growth, the trend (in growth) is even declining*…’ (BB, industry association)
2019	On tobacco tax ‘…*one of the goals of increasing cigarette excise is to reduce cigarette consumption among children*…’ (HP, MoF officials)	
2020	On SSB tax ‘…*if this [SSB tax] is to be imposed, we will receive an additional revenue of IDR 6.25 trillion*…’ (SMI, MoF officials)	On tobacco tax ‘*…the increase in tax on tobacco products would decrease the state revenues because people with low purchasing power would switch to illegal or cheaper products…’* (HN, industry association) On SSB tax ‘…*any factor resulting in the industry’s difficulties must be eliminated. Whether it concerns imported raw materials, product exports, or fiscal regulations, we are doing our best to help our domestic industry*…’ (AGK, government officials from Ministry of Industry/MoI)
2022	On SSB tax ‘…*many companies have reformulated their products by gradually decreasing the sugar level on their products and educate the consumers to choose healthier alternatives*…’ (ASL, industry associations)	

SSB, sugar-sweetened beverage.

**Table 2:** Health tax policy dynamics in Indonesia

Timeline	Policy milestone
15 Aug 2007*	Law No.39/2007 on amendments of Law No. 11/1995 on Excise
9 Dec 2008†¶	7% increase in tax tariff on tobacco products (Minister of Finance Regulation (PMK) No. 203/PMK.011/2008)
15 Sep 2009* **	Law No. 28/2009 on Regional Tax and Retribution. The regional government is entitled to a cigarette tax (paid by the consumers) and alcoholic beverages retribution (paid by sellers)
3 Dec 2009†¶	PMK No. 197/PMK.07/2007 on the Guideline for The Distribution of Tobacco Tax Sharing Fund to Tobacco Products and Tobacco Producing Provinces
17 Mar 2010†	PMK No. 62/PMK.011/2010 on Tax Rates for Ethyl Alcohol, Beverages Containing Ethyl Alcohol and Concentrates Containing Ethyl Alcohol
31 Dec 2013†**	The implication of PMK No. 207/PMK.011/2013: increasing tax tariff for alcoholic beverages, especially for imported products
6 Nov 2015‡	PMK on Tobacco Tax Increase in 2016 (PMK No. 198/PMK.10/2015). Average increase of 11.19%
2016§	The tax duty on SSB was proposed for the first time
30 Sep 2016‡	PMK on Tobacco Tax Increase in 2017 (PMK No. 147/PMK.010/2016). Average increase of 10.54%
25 Oct 2017‡	PMK on Tobacco Tax Increase in 2018 (PMK No. 146/PMK.010/2017). Average increase of 10.04%. Planning the simplification of tobacco tax tariff (from 10 to 8 to 6 to 5 tiers in 2018, 2019, 2020 and 2021, respectively)
1 Oct 2018†**	Tax tariffs on vape products (PMK No. 146/PMK.010/2017). Should have been effective on 1 July 2018, but were delayed to 1 October 2018, due to the regulatory relaxation
12 Dec 2018†**	The implication of PMK No. 156/PMK.010/2018: (1) no tax increase on tobacco in 2019; (2) a roadmap of simplification of tax tariffs on tobacco products was removed and (3) tax duty on electric cigarette products were applied (57% of the retail price).
12 Dec 2018†**	The implication of PMK No. 158/PMK.010/2018: increasing tax tariff for alcoholic beverages, especially for imported products
18 Oct 2019†**	PMK on Tobacco Tax Increase in 2020 (PMK No. 152/PMK.010/2019). Average increase of 23%
Early 2020§	SSB tax proposal was mentioned again in a joint meeting between Commission XI of the People’s Consultative Assembly and the Minister of Finance (proposed by MoF)
14 Dec 2020‡	PMK on Tobacco Tax Increase in 2021 (PMK No. 198/PMK.010/2020). Average increase of 12.05%
29 Oct 2021*	Law No. 7 of 2021 on Tax Harmonisation loosens the process of extending the taxable goods
17 Dec 2021‡	PMK on Tobacco Tax Increase in 2022 (PMK No. 192/PMK.010/2021). Average increase of 12%
20 Dec 2021‡	PMK No. 193/PMK.010/2021 concerning Tax Rates on Heated Tobacco Products. E-cigarette tax increase by 17.5%
End of 2021§	The plan to implement tax duty on SSB in 2022
March – April 2022§	Postponement of SSB tax policy to 2023

*Policy related to health taxes in general.

†Policy related to tobacco products tax.

‡Policy related to alcoholic beverages tax.

§Policy related to SSB tax.

¶Policy signed pre-election (up to a year before).

**Policy signed postelection (less than a year after).

PMK, Peraturan Menteri Keuangan; SSB, sugar-sweetened beverage.

During election years (eg, up to a year pre-election and less than a year postelection), the health tax policies, primarily on tobacco taxes, were modified. During the election of 2009, there was no significant change pre-election year. However, we noticed a shift in tax decentralisation less than a year postelection. In the election year of 2014, there were no tobacco tax tariff increases a year preelection and postelection. In 2013 (pre-election year), there was only an increase in tariff for alcoholic beverages, especially for imported products.

In the 2019 election, the health tax policies have been changing considerably, particularly pre-election year. First, there was no increase in tobacco tax tariff in 2018. The absenteeism of the increase in tariff in the pre-election year of 2018 could be explained by the policy dynamics in tobacco tax. More political figures commented on tobacco tax in pre-election years—most opposing health taxes (see online supplemental appendix A1). Second, e-cigarette tax was implemented for the first time but had been delayed by 4 months due to relaxed regulations. Third, alcoholic beverages tax tariff was increased for the first time since 2013, particularly on imported products. Finally, the 2019 proposal for SSB tax was not discussed further until 2021.

10.1136/bmjgh-2023-012042.supp1 Supplementary data

To discuss the differences among actors, news sentiments and statements for each commodity, we summarised the number of news articles by the timeline, news tone, top 10 actors cited and statement’s theme (table 3 and table 4). The majority of news articles covered tobacco tax policies (N=9940), while the news coverage on alcoholic beverages (N=242) and SSB (N=241) were significantly less. This is consistent with the findings in table 2, whereby tobacco encountered more policy changes than alcoholic beverages and SSB.

**Table 3:** Share of news article by commodity type, timeline and news tone, 1 January 2018–3 July 2022

Timeline	Tobacco (N=9940)			% of News by Time	Alcoholic beverages (N=242)			% of News by Time	SSB (N=241)			% of News by Time
	% Neutral (N=3378)	% Positive (N=2446)	% Negative (N=3616)		% Neutral (N=55)	% Positive (N=59)	% Negative (N=108)		% Neutral (N=120)	% Positive (N=61)	% Negative (N=62)	
Q1 2018	27	33	40	2	100	0	0	0.5	100	0	0	0.8%
Q2 2018	31	38	31	2	100	0	0	0.5	N/A (n=0)	N/A (n=0)	N/A (n=0)	N/A (n=0)
Q3 2018	36	32	33	3	0	100	0	0.5	N/A (n=0)	N/A (n=0)	N/A (n=0)	N/A (n=0)
Q4 2018	42	27	31	5	40	0	60	2	N/A (n=0)	N/A (n=0)	N/A (n=0)	N/A (n=0)
Q1 2019	37	23	40	1	6	24	71	8	N/A (n=0)	N/A (n=0)	N/A (n=0)	N/A (n=0)
Q2 2019	34	32	34	1	23	23	54	6	N/A (n=0)	N/A (n=0)	N/A (n=0)	N/A (n=0)
Q3 2019	34	23	43	10	55	18	27	5	N/A (n=0)	N/A (n=0)	N/A (n=0)	N/A (n=0)
Q4 2019	32	25	42	10	13	20	67	7	N/A (n=0)	N/A (n=0)	N/A (n=0)	N/A (n=0)
Q1 2020	31	30	39	6	5	37	58	9	48	26	26	35%
Q2 2020	27	34	39	3	17	67	17	3	0	0	100	0.4%
Q3 2020	31	30	39	6	33	33	33	3	50	0	50	0.8%
Q4 2020	42	21	37	15	26	18	56	15	100	0	0	0.4%
Q1 2021	36	28	36	4	24	10	67	9	60	10	30	4%
Q2 2021	24	29	47	4	25	33	42	11	78	11	11	4%
Q3 2021	37	21	42	10	40	30	30	5	39	33	28	7%
Q4 2021	43	22	36	11	39	36	25	13	54	23	23	11%
Q1 2022	33	35	32	4	0	17	83	3	36	40	24	10%
Q2 2022	36	29	35	3	0	75	25	2	51	23	26	27%
% of news by tone	36	26	38	% of news by tone	25	27	49	% of news by tone	49	25	26	

SSB, sugar-sweetened beverage.

**Table 4:** Share of news article from top 10 most cited actors by commodity type and theme, 1 January 2018–3 July 2022

Commodities	Key opinion leaders (top 10 most cited actors)	Public health	Industry	Politics	Economy
Tobacco Statements (top 10): N=6065 14% public health; 35% industry; 6% politic; 44% economy Actors: 3 MoF officials; 2 NGOs/experts; 2 politicians/other governmental figures (except MoF); 3 industry proponents	Sri Mulyani Indrawati (SMI)†¶** (n=1550)	14%	29%	6%	51%
	Henry Najoan* (n=940)	10%	50%	2%	39%
	Agus Parmuji* (n=691)	5%	41%	2%	52%
	Heru Pambudi (HP)†¶ (n=564)	11%	32%	4%	53%
	Joko Widodo† (n=485)	18%	32%	6%	43%
	Tulus Abadi (TA)‡** (n=479)	43%	16%	14%	28%
	Sulami Bahar* (n=459)	7%	51%	2%	40%
	Mukhamad Misbakhun† (n=320)	7%	31%	28%	34%
	Abdillah Ahsan (AA)‡** (n=345)	26%	26%	3%	45%
	Nirwala Dwi Heryanto (NDH)†¶ (n=232)	13%	36%	8%	43%
Alcoholic beverages Statements (top 10): N=476 10% public health; 18% industry; 3% politic; 69% economy Actors: 4 MoF officials; 2 NGOs/experts; 2 politicians/other governmental figures (except MoF); 2 industry proponents	Bhima Yudhistira Adhinegara‡ (n=107)	21%	14%	1%	64%
	Sarman Simanjorang* (n=72)	13%	46%	13%	29%
	Fransiskus Xaverius Hadi Rudyatmo† (n=53)	11%	0%	0%	89%
	NDH††† (n=49)	6%	31%	0%	63%
	Budi Santoso† (n=43)	0%	0%	0%	100%
	Syarif Hidayat§ (n=39)	0%	18%	5%	77%
	SMI†††¶ (n=36)	0%	6%	8%	86%
	HP††† (n=29)	0%	10%	0%	90%
	Bambang Britono* (n=25)	4%	32%	0%	64%
	Pingkan Audrine Kosijungan ‡ (n=23)	22%	4%	4%	70%
SSB Statements: N=719 16% public health; 13% industry; 5% politic; 67% economy Actors: 3 MoF officials; 4 NGOs/experts; 1 politician/other governmental figures (except MoF); 2 industry proponents	Sri Mulyani Indrawati (SMI)†††¶ (n=192)	17%	2%	12%	69%
	Askolani§ (n=156)	0%	0%	5%	95%
	Adhi S Lukman* (n=119)	18%	20%	1%	61%
	Triyono Prijosoesilo* (n=67)	9%	49%	1%	40%
	Febrio Nathan Kacaribu§ (n=47)	43%	0%	4%	53%
	AA‡†† (n=40)	33%	3%	5%	60%
	Fithra Faisal Hastiadi‡ (n=37)	19%	11%	0%	70%
	Agus Gumiwang Kartasasmita† (n=23)	0%	70%	0%	30%
	Michael Wilson Setjoadi‡ (n=21)	0%	38%	0%	62%
	TA‡†† (n=17)	65%	0%	0%	35%

*Industry proponents.

†MoF officials.

‡Politician or other governmental figures (except MoF).

§NGOs or experts.

¶Overlapping actors with alcoholic beverages tax.

**Overlapping actors with tobacco tax.

††Overlapping actors with SSB tax.

MoF, Ministry of Finance; NGO, nonprofit organization; SSB, sugar-sweetened beverage.

We identified 25 actors whose statements are most frequently cited by the media from January 2018 to July 2022. Five key actors (three MoF officials and two NGOs/experts) overlap for different commodities (eg, Sri Mulyani Indrawati (SMI), Abdillah Ahsan (AA), Tulus Abadi (TA), Nirwala Dwi Heryanto (NDH), Heru Pambudi (HP)). There were no overlapping politicians and industry proponents across all commodities. SMI, as the current MoF, was featured as one of the top 10 most cited actors in all commodities. However, she was not the main figure in the news on alcoholic beverages. At least three MoF officials are the top 10 most cited actors for all commodities. However, the industry-related statements on tobacco tax were more prominent among MoF officials—compared with the other two commodities.

More news coverage’s share on public health-related statements in SSB tax (16%) is an important opportunity for health tax implementation. MoF’s officers’ statements had more public health-themed arguments in SSB tax than the other two commodities. MoF officials featured across all commodities, indicating policies are moving in the intended direction with MoF as the main actors in health tax implementation.

### Tobacco tax

Each year, most news articles covering tobacco tax emerged in the third and fourth quarters (see table 3). This is primarily due to the government announcement of an annual increase in tobacco products tax since 2015. However, since there was no increase in tariff for 2019 announced in 2018 (pre-election year), the news coverage during the third and fourth quarters of 2018 was minimum (compared with the same period in another year) (see online supplemental appendix A2). The industry interference during this period could also be illustrated by the share of negative articles during the third and fourth quarters of each year (table 3).

The industry interference in tobacco tax is also shown by more industry proponents as the most cited actors in the media—compared with two other commodities. Three industry proponents (eg, Henry Najoan (HN), Agus Parmuji (AP) and Sulami Bahar (SB)) are key opinion leaders. HN and AP were frequently cited in the media, ranked second and third after SMI (current MoF). They even topped the current President of Indonesia, Joko Widodo (JW) and two other MoF officials (HP and NDH). The politicians’ statements on tobacco tax (JW and Mukhamad Misbakhun (MM)) are more inclined towards industry than public health. Statements favouring public health emerged from NGOs/experts (TA and AA). Lack of media coverage for public health statements (14% of total top 10 key opinion leaders statements) compared with industry-themed statements (35%) became a challenge—particularly during political years.

The absence of a tax increase in 2019 illustrated the strong tobacco industry interference and political lobbying. This is also illustrated in most of the counter statements from politicians arguing for the giant tobacco industry, hiding behind the welfare of retailers, tobacco farmers and industry workers. One of the politicians (MM) argued that ‘the policy had been made based on the aspirations of retailers, industry and tobacco farmers’.

After the 2019 public election, MoF support towards tobacco products tax was more profound as SMI became the most frequently cited key opinion leader throughout 2020–2022 (see online supplemental appendix A1). Her statements were majorly on economic themes, that is, revenue generation. This is also true for other MoF officials (HP and NDH). While still few, the MoF officials’ awareness of the importance of tobacco tax on public health implies opportunities for tobacco taxation in Indonesia. This is illustrated in the statements of HP (MoF official): ‘One of the goals of increasing cigarette excise is to reduce cigarette consumption among children’.

After the election year, more opposing arguments towards tobacco products came from the industry proponents—instead of politicians (online supplemental appendix A1). During the economic downturn due to COVID-19, there are emerging misleading statements on the harm of tobacco tax for illicit cigarette sales from these actors. HN from the industry association stated that ‘the increase in tax on tobacco products would decrease the state revenues because people with low purchasing power would switch to illegal or cheaper products’.

### Alcoholic beverages tax

The implementation of the alcoholic beverages tax lacked political interests as there were fewer politicians and other governmental figures (two persons) giving their opinion on the issue—compared with four persons from MoF officials (see table 4). The statements from NGOs/experts (Bhima Yudhistira Adhinegara, contributing to 22% of the total top 10 actors statements) ranked first, exceeding those from industry proponents, MoF officials and politicians/other governmental actors. Within the same period of tobacco tax, there were significantly fewer articles released in the media. The policy change for alcoholic beverages in 2018 (ie, the increase of 15% tariff in alcoholic beverages category A (containing up to 5% ethyl alcohol) was not even widely covered in the media (see table 3). Furthermore, 4 of the 10 most cited actors in the media were from MoF officials themselves (32% of total statements of the top 10 were from MoF officials). This indicated fewer political and industry interests to interrupt the policy-making process—compared with tobacco taxation.

Although the majority of the news tone was more negative on the alcoholic beverages tax issue (up to 49%), they did not widely feature in industry-related themes (only 18%). Majority of statements in alcoholic beverages concerned economic theme, that is, revenue generation. Generally, the increase in alcoholic beverages tax in 2018 was supported by NGOs and experts because there has been no increase in its tariff since 2013 (see table 1). Since 2013, the government has never evaluated alcohol tax tariffs, even though it is necessary to consider inflation and affordability. Furthermore, the level of alcoholic beverage production before 2018 was steady, as stated by NDH (MoF official): ‘Although there was no increase in (alcoholic beverages) production for 4 years, there was also no increase in tax duty. Therefore, the tax payments to factory turnover ratio decreased in the same period’.

The only opposing arguments came from the industry association (BB), arguing that sales of alcoholic beverages have been decreasing and therefore increasing tariff policy will be a disadvantage to the industry: ‘since 2014–2017 and also 2018, the domestic industry for (alcoholic beverages) type A or beer has a steady growth, the trend (in growth) is even declining’. In the fourth quarter of 2020, there was a sudden increase in the number of news articles with negative tone statements (see online supplemental appendix A3) following the issue of the law to prohibit the sales of alcoholic beverages.

### SSB tax

SSB taxation is still under government review and is planned for implementation in 2023. It was first proposed in 2018 but only publicly announced in the first quarter of 2020. The media nearly uncovered the first proposal in 2018 (see table 3). During this period, the articles mostly covered the MoF’s announcement to impose a tax on SSB. However, the issues disappeared significantly throughout the second to fourth quarters of 2020. The issue only reemerged in the second quarter of 2021 and continued to increase throughout 2022. The negative sentiments towards the issue increased at times when the tax imposition was announced (see online supplemental appendix A4). As discussed earlier, the rigid regulatory structure often demanded a regulation to come first before a multisectoral policy discussion. The Law on Tax Harmonisation in 2021 (see table 2) allows an easier process of extending taxable goods. This facilitates the policy discussion as MoF now has the basis for taxing SSB, which previously was not a taxable good.

During 2020–2022, the most frequently cited actors were similar; hence we summarised the key opinion leaders throughout this period (table 4). Like tobacco products, the current MoF, SMI, was the most frequently cited in SSB tax. Most of her statements (69%) concerned economic issues and revenue generation: ‘If this (SSB tax) is to be imposed, we will receive an additional revenue of IDR 6.25 trillion’. This was followed by other MoF officials, AS (95% statements on economic themes), and Febrio Nathan Kacaribu (FNK) (53% statements on economic themes).

SMI’s takes on SSB tax and public health (17% of total statements) will be important opportunities in SSB tax implementation. This is also consistent with other MoF officials, FNK (43% statements on public health themes). The 2022 government’s announcement to impose SSB tax by 2023 led to different NGOs/experts’ opinions. While two public health experts were supportive of the plan (eg, TA and AA), the economic analyst was either neutral (Fithra Faisal Hastiadi) or unsupportive (Michael Wilson Setjoadi). The industry proponents (Adhi S Lukman (ASL) and Triyono Prijosoesilo) firmly argued that the SSB tax would not impact public health. The arguments to protect the industry also come from other government officials (Agus Gumiwang Kartasasmita, the Ministry of Industry), particularly due to the pandemic: ‘Any factor resulting in the industry’s difficulties it must be eliminated. Whether it concerns imported raw materials, product exports, or fiscal regulations, we are doing our best to help our domestic industry’.

Intriguingly, the arguments from the industry proponent have been compromised in the following years. They believed that product reformulation would be more important in affecting public health. They further argued that the industries had taken promotive measures by educating the consumers to switch to healthier products. As ASL (an actor from the industry association) stated in 2022, ‘Many companies have reformulated their products by gradually decreasing the sugar level on their products and educating the consumers to choose healthier alternatives’.

## Discussion

The political economy of health taxes in Indonesia outlined four main actors’ classifications, for example, the MoF officials, industry proponents, NGOs/experts and politicians or non-MoF government officials. Each category of actor holds a different view in the policy arena. NGOs/experts and MoF officials are more likely to support health taxes in the media. They opposed the views of politicians, non-MoF government officials and industry proponents. The polarised debate is tied to the unique context of political arrangement in Indonesia: multiparty system and regional autonomy.

The technocracy in Indonesia has been nurtured since the New Order regimen under President Soeharto.^35^ Technocrats held key ministerial positions in economics until the post-Soeharto era (also known as the reform era).^35^ The first presidential election of 2004 set Indonesia to be the world’s largest multiparty presidential democracy.^36^ This has significantly changed the political landscape. Choosing the economic team of the ministers becomes highly sensitive for interparty support.^37^ From all the strategic economic ministers, only MoF is led by technocrats today, while other key economic teams of the ministers are given to the politicians from the winner’s party’s coalition. This explained the views of MoF (proponents) and non-MoF (mostly opponents) on health taxes. Having technocrats as MoF also explained the agency’s health taxes support in favour of revenue generation and the insufficient public health arguments.

An open competition for a multiparty system also creates a demand and supply for political support from the industry. This creates ‘political entrepreneurs’ through two activities: rent-seeking and rent extraction to gain campaign sponsorship from the industry.^38^ This is particularly applied to politicians from mass parties where the party’s financing comes from the members.^39^ This explained the politicians’ opponents’ views during election years, particularly tobacco taxes. The strong political lobby from the giant transnational tobacco company is also evidenced in other ASEAN LMICs. The industry is either directly approaching the dominant party or lobbying the susceptible ministers/politicians in a multiparty country.^40 41^ This approach is applied globally as an organised tobacco industry tactic under Concordia, gathering strategic leaders and policy-makers in defending the industry.^42^

The complex policy process in tobacco is tightly related to the sizeable market share. Indonesia is still the largest market for cigarette consumption among male adults and is overly normalised among adolescents through massive tobacco advertising, promotion and sponsorship.^14 43^ The industry’s power to approach political leaders outweighed those of public health supporters.^44 45^ This could be a challenge for SSB tax as it possesses similar industry characteristics—a giant size to support the political lobby. This has been presented by the Ministry of Industry’s statements to protect the enterprises strongly.

The decentralised governmental system also plays an important role in disentangling the unique political context for health tax implementation in Indonesia. Regional autonomy increases the political power of local politicians. It again creates a demand and supply for the industry’s political sponsor. Furthermore, the combination of Indonesia’s rigid hierarchy of regulatory structure and regional autonomy results in weak implementation at the local level.^44^ In some parts where the industry is dominant (for instance, in East Java for tobacco commodities), it is extremely challenging to gain support from the local government and communities.^46^

Both multiparty systems and regional autonomy also lead to a complex bureaucracy and a lack of intersectoral coordination.^44^ The rigidly divided authority has also resulted in the exclusion of some potential allies, such as the Ministry of Health (MoH). The role of MoH has also been limited as there is insufficient media coverage for MoH’s takes on health taxes. The MoH focuses more on the non-fiscal measures of NCD control. In addition, MoH has become the sole champion in favouring public health interests.^44^

The discussion above showcases the three main challenges for health tax policy media debates: (1) policy contestation, (2) elections and (3) lack of public health supporters (particularly MoH) exposure in the media.

Due to the divided views resulting in the political context, the policy contestation among different government institutions on tobacco and SSB taxation becomes the first major challenge. This study implied that the health tax for the two commodities had become a constant debate between a coalition of proponents (MoF and public health supporters) and opponents (the politicians in the ministries and legislative). In other countries, government officials commonly allied with health tax advocates to raise public awareness of health issues.^47–49^ This is particularly profound when public health benefits become the basis for policy advocates.^50^ The political structures with rigid hierarchy, lack of respect towards MoH and the complex government bureaucracy are serious institutional barriers to fully adopting the health tax policy.^44^

Another challenge for implementation is elections. As presented by tobacco taxation in 2018, there will be increasing anti-health tax arguments (primarily from politicians) using ‘vulnerable groups’ to support the industry. This practice is also common in other countries.^33 51^ While there was enough pro-health tax evidence against the industry’s arguments,^52–55^ these studies were rarely covered in the media. The lack of media interest in covering scientific evidence in opposing the industry is unfortunately related to the well-funded industry in commissioning the media.^44^

On the other hand, the media coverage favouring health tax during the political years could be an advocate to control consumers’ purchasing behaviour.^51 56^ This is particularly true for media framing using public health promotion. Other countries’ experiences implied that rising arguments favouring public health could be used as media framing strategies for health taxes, particularly during elections.^51^ Generating evidence to compromise ‘vulnerable groups’—in addition to public health—is also recommended.^57^

The main opportunity for health taxes is the support from MoF’s officials, the leading actor for health tax implementation based on the regulatory structure and their arguments in the media. MoF creates and implements the health tax policy. Drawing from the SSB taxation findings, most of MoF’s officials favoured public health. This increases news articles on health issues supporting health taxes, as MoF’s officials are the most frequently cited opinion leaders. Particularly, this should be adopted by tobacco tax advocates. Generating evidence, continuing capacity-building, linking health to finance and sharing international best practices that favour revenue generation and public health goals, will be important strategies to advocate for the MoF’s officials in health taxes passages.^58–60^ Expanding the training and fostering the allies with government officials of industry proponents (eg, Ministry of Industry) also remain critical to challenge the industry’s strategy.^61^

Drawing from alcoholic beverages experience, small market size is associated with a lack of political interests and industry interference. Religious reason becomes the main factor for the niche market. This could be an important lesson learnt for tobacco tax. There has been a constant debate on the prohibition of tobacco consumption among Islamic scholars in Indonesia.^62^ Generating allies with religious public figures and adopting the tobacco prohibition will be significant in reducing consumption and avoiding further policy debates.^62 63^

This study is the first to document the media coverage across key health taxes commodities in Indonesia. This study also provides a solid political contextualisation to understand the challenges and opportunities facing health taxes implementation in Indonesia. The study’s methodological sections used general terminologies in the search strategy, allowing for replication in countries with comparable health tax regulations. However, this study is subject to several limitations due to data availability. First, our data was limited to begin from 2018—while tobacco and alcohol taxes were passed decades earlier. Future studies should extend the timeline coverage to understand the policy dynamics, especially during political years. Second, the media covers significantly fewer articles on alcohol and SSB taxation compared with tobacco taxation. Third, as only Indonesian-language articles were included, this limited our articles’ coverage to only national media. We suggest further studies to engage international articles. Fourth, our study is limited to news coverage. Future studies should include quotations in social media.

To account for the four limitations, we contextualised, reflected and confirmed the findings with our main author’s (AA) expertise in the health policy context, as he was featured prominently in SSB and tobacco tax. Finally, there are few local governments cited in the national media. Therefore, further study should investigate local governments’ takes on health taxes.

## Conclusion

This paper aims to investigate the policy dynamics of health taxes by exploring the media coverage of alcohol, SSB and tobacco taxes in Indonesia. We focused on the key actors, timeline and the topic of the arguments of each commodity. A complex political process in health tax implementation was found in tobacco tax. The policy tense raised during the pre-election years. While the issue of SSB taxation is emerging, the key opinion leaders’ statements in the media imply ongoing policy contestation. Public health support was gained mostly from NGOs/experts, while MoH lack of media exposure. MoI and politicians are protecting the industry. MoF officials are the most cited actors, with views inclining towards economic benefits. However, the MoF’s commitment as the leading actor on health taxes should be taken as the main opportunity in health taxes passage.

The political arrangement in Indonesia creates three major challenges against health tax implementation. First, the policy contestation due to the political setting. The second challenge is the election years, resulting in some anti-health tax arguments during these periods. Finally, the media’s lack of arguments in favour of public health remains a challenge. While MoF shows positive-toned arguments towards health taxes, they are mainly framed on revenue generation. With the media’s lack of coverage of MoH, the argument favouring public health is insufficient.

Generating public allies with key religious opinion leaders is important to address the first and second challenges. The lack of policy contestation for alcohol taxes can be attributed to the strong religious prohibition on alcohol consumption. Encouraging similar social prohibitions against tobacco use could result in stronger tobacco control policies and increased public support for tobacco control initiatives.

Public coalitions should also identify potential politicians for allies. Continuing capacity building on politicians with opposing views towards public health is recommended. To address the third challenge, gathering evidence in favour of public health, revenue generation and ‘vulnerable groups’ protection to support the MoF is recommended. Other LMICs found that showcasing evidence-based arguments on the health benefits of health taxes gained more public support. In addition, continuing capacity-building to support the MoH in gaining media exposure on health taxes is important.

Future studies should engage international articles and include social media analysis. Further research is needed to investigate the local governments’ takes on health taxes.

## Data Availability

Data may be obtained from a third party and are not publicly available.
