# Supplementary material for: Health taxes in Indonesia: a review of policy debates on the tobacco, alcoholic beverages and sugar-sweetened beverage taxes in the media
Source: BMJ Glob Health. 2023 Oct 9;8(Suppl 8):e012042. doi: 10.1136/bmjgh-2023-012042 (PMC10565181; doi:10.1136/bmjgh-2023-012042)
Supplement: Supplementary data [file bmjgh-2023-012042supp001.pdf]

Appendix A1 Top 10 most cited actors in news articles for tobacco products tax in Indonesia, 2018 – 2022

2018	2019	2020	2021	2022
Tulus Abadi[*,p]	Heru Pambudi[†,n]	Sri Mulyani Indrawati[†,p]	Sri Mulyani Indrawati[†,p]	Sri Mulyani Indrawati[†,p]
Heru Pambudi[†,n]	Tulus Abadi[*,p]	Henry Najoan[¶,c]	Henry Najoan[¶,c]	Agus Parmuji[¶,c]
Abdillah Ahsan[*,p]	Henry Najoan[¶,c]	Agus Parmuji[¶,c]	Tulus Abadi[*,p]	Mayagustina Andarini[§,p]
Yustinus Prastowo[*,p]	Suhariyanto[§,n]	Sulami Bahar[¶,c]	Mukhamad Misbakhun[§,c]	Anis Byarwati[§,p]
Mukhamad Misbakhun[§,c]	Joko Widodo[§,p]	M. Al Khadziq[§,c]	Faisal Basri[*,p]	Sulami Bahar[¶,c]
Sri Mulyani Indrawati[†,n]	Agus Parmuji[¶,c]	Heru Pambudi[†,n]	Sulami Bahar[¶,c]	Tulus Abadi[*,p]
Airlangga Hartarto[§,c]	Enny Sri Hartati[*,c]	Heri Susanto[*,c]	Firman Soebagyo[§,c]	Firman Soebagyo[§,c]
Bambang Haryo Soekartono[§,c]	Dita Indah Sari[§,c]	Tulus Abadi[*,p]	Nirwala Dwi Heryanto[†,n]	Henry Najoan[¶,c]
Muhammad Jusuf Kalla[§,n]	Sulami Bahar[¶,c]	Nirwala Dwi Heryanto[†,n]	Dante Saksono Harbuwono[§,p]	Agus Suyatno[*,p]
Agus Parmuji[¶,c]	Abdillah Ahsan[*,p]	Abdillah Ahsan[*,p]	Hananto Wibisono[¶,c]	Faisal Basri[*,p]

* NGOs or experts
† Ministry of Finance (MoF) officials
§ Political figures or other governmental officials (except MoF)
¶ Industry proponents (industry associations or tobacco farmers)
p Statements are mostly pros toward tobacco tax
c Statements are mostly cons toward tobacco tax
n Statements are mostly neutral toward tobacco tax

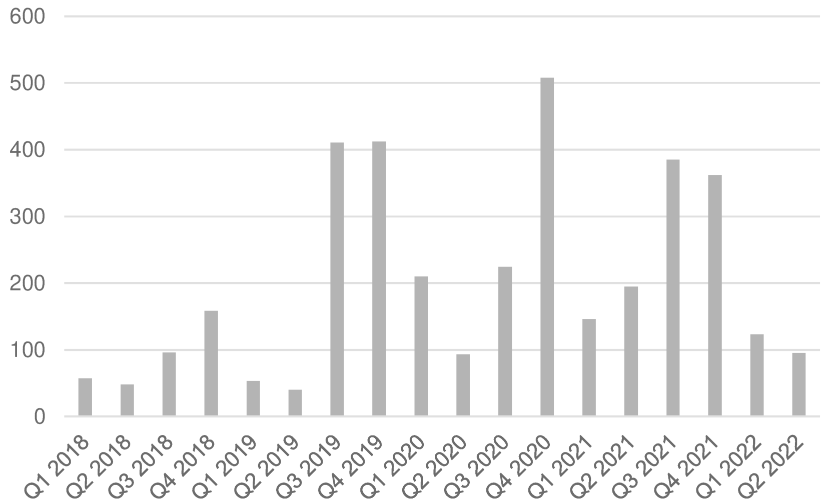

Appendix A2 Quarterly Trends in Number of Tobacco Tax News with Negative Sentiments, Indonesia 2018 – 2022

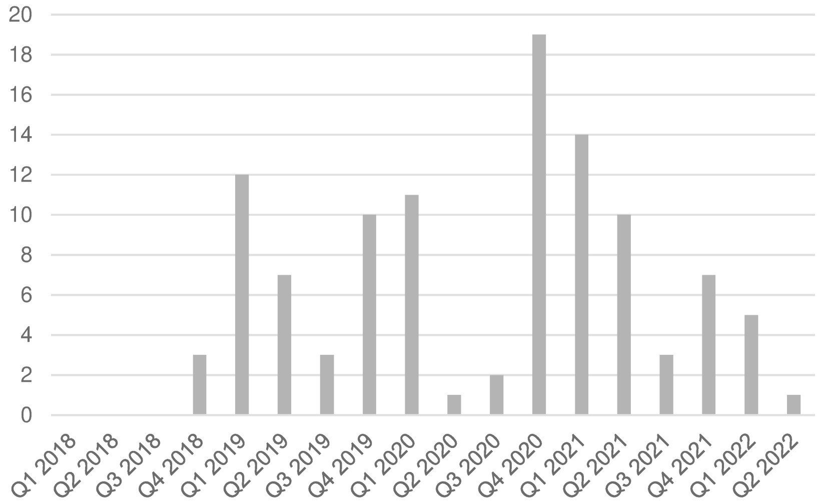

Appendix A3 Quarterly Trends in Number of Alcohol Tax News with Negative Sentiments, Indonesia 2018 – 2022

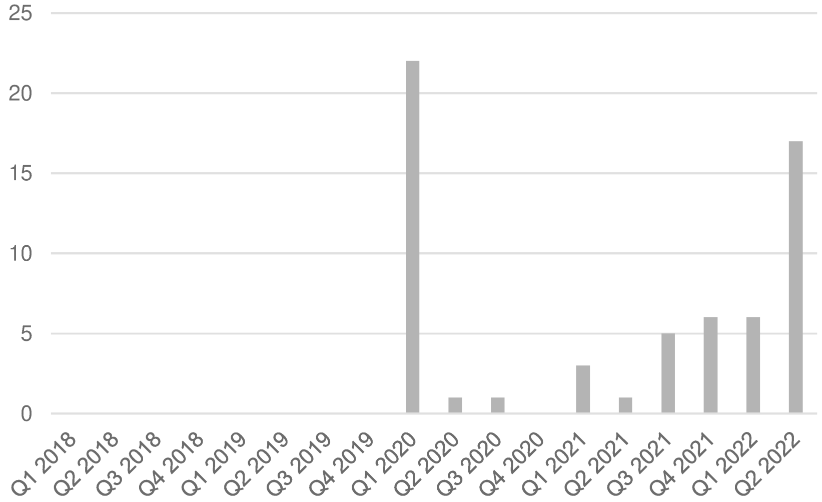

Appendix A4 Quarterly Trends in Number of SSB Tax News with Negative Sentiments, Indonesia 2018 – 2022
